# Compassion protects against vital exhaustion and negative emotionality

**DOI:** 10.1007/s11031-021-09878-2

**Published:** 2021-04-13

**Authors:** Aino Saarinen, Liisa Keltikangas-Järvinen, Essi Viding, Henrik Dobewall, Kaisa Kaseva, Terho Lehtimäki, Olli Raitakari, Mirka Hintsanen

**Affiliations:** 1 Research Unit of Psychology, University of Oulu, Oulu, Finland; 2 Department of Psychology and Logopedics, Faculty of Medicine, University of Helsinki, Helsinki, Finland; 3 Division of Psychology and Language Sciences, University College London, London, UK; 4 Cicero Learning, Faculty of Educational Sciences, University of Helsinki, Helsinki, Finland; 5 Department of Clinical Chemistry, Fimlab Laboratories, and Finnish Cardiovascular Research Center, Tampere, Finland; 6 Faculty of Medicine and Health Technology, Tampere University, Tampere, Finland; 7 Centre for Population Health Research, University of Turku and Turku University Hospital, Turku, Finland; 8 Research Centre of Applied and Preventive Cardiovascular Medicine, University of Turku, Turku, Finland; 9 Department of Clinical Physiology and Nuclear Medicine, Turku University Hospital, Turku, Finland

**Keywords:** Compassion, Personality, Stress, Psychosocial stress, Longitudinal

## Abstract

We investigated (i) the predictive relationships of compassion with negative emotionality (a marker of susceptibility to stress) and vital exhaustion (a marker of chronic stress response) and (ii) the effect of compassion on the developmental courses of negative emotionality and vital exhaustion over a follow-up from early adulthood to middle age. We used the prospective Young Finns data (*n* = 1031–1495, aged 20–50). Compassion was evaluated in 1997, 2001, and 2012; and vital exhaustion and negative emotionality in 2001, 2007, and 2012. The predictive paths from compassion to vital exhaustion and negative emotionality were stronger than vice versa: high compassion predicted lower vital exhaustion and lower negative emotionality. The effect of high compassion on lower vital exhaustion and lower negative emotionality was evident from early adulthood to middle age. Overall, high compassion appears to protect against dimensions of stress from early adulthood to middle age, whereas this study found no evidence that dimensions of stress could reduce disposition to feel compassion for others’ distress over a long-term follow-up.

**Supplementary Information:**

The online version contains supplementary material available at 10.1007/s11031-021-09878-2.

## Introduction

The widely used classification for mental disorders, DSM-5 (Diagnostic and Statistical Manual of Mental Disorders, 5th Edition), lists stress responses as a crucial trigger for almost all psychiatric disorders (American Psychiatric Association (APA), 2013). Additionally, stress responses also predisposes to the incidence of a wide range of somatic diseases, such as coronary heart disease (Steptoe & Kivimäki, 2012), cancer (Chida et al., 2008), inflammatory diseases (Black, 2002), and gastrointestinal disorders (Mönnikes et al., 2001). Taken together, stress responses compose a major risk for both mental and somatic health.

Consequently, a wide variety of stress-reducing interventions have been developed, ranging from practical life-style interventions (Merrill et al., 2007) to cognitive-behavioral therapies and hypnotic treatments (Bryant et al., 2005). In the recent years, an increasing amount of research has been directed to the development of compassion-cultivating interventions aiming to reduce stress (e.g. Abelson et al., 2014; Gilbert, 2014; Jazaieri et al., 2013). By definition, compassion includes an affective component (i.e. feeling concern for other’s distress) and a motivational component (i.e. the desire to alleviate other’s distress) (Gilbert, 2019; Goetz et al., 2010).

Stress can be measured from a variety of perspectives: amount of exposure to stressors, susceptibility to stress responses, or actualized stress responses (Epel et al., 2018). Stress responses, in turn, can refer to psychological, physiological, or neural responses and to short-term (acute) or long-term responses (chronic responses leading to allostatic load) (Epel et al., 2018). In this study, we aimed to investigate the relationships of compassion with two stress-related dimensions: vital exhaustion and negative emotionality. Vital exhaustion refers to a marker of chronic stress responses (Koertge et al., 2002). High vital exhaustion is found to associate with altered cardiac reactivity to task-induced stress (Keltikangas-Järvinen & Heponiemi, 2004), reduced habituation of free cortisol responses to repeated acute psychosocial stress (Kudielka et al., 2006), increased s-prolactin (Halford et al., 2003), and altered cortisol levels and higher perceived stress (Nicolson & van Diest, 2000). Hence, vital exhaustion refers to chronically elevated stress responses that remain despite there would not be any current (acute) exposure to stress. Negative emotionality, in turn, is a marker of dispositional susceptibility to stress, i.e. a relatively stable personality trait predisposing to elevated stress levels in response to even comparatively mild environmental stressors (Buss & Plomin, 1984; Ebner & Singewald, 2017; Strelau & Angleitner, 1991). There is evidence that high negative emotionality predicts higher stress at work (Hintsanen et al., 2011), elevated cortisol levels in response to conflicts (Laurent & Powers, 2007), and higher depression and anxiety (Skipstein et al., 2012).

There are several theoretical models about the relationship between compassion and stress responses. Firstly, compassion includes a non-judgemental attitude toward others’ negative affective states that is proposed to simultaneously enhance affective regulation and tolerance of one’s own distress (Gilbert, 2019; Kirby & Gilbert, 2017). Secondly, feelings of social belongingness are suggested to compose a fundamental evolutionary need for the human being (Brown & Brown, 2017). A lack of compassionate feelings may interfere with forming and maintaining balanced social relationships and cause distress via social rejection and interpersonal conflicts (Brown & Brown, 2017). Thirdly, it has been postulated that compassionate inter-dependence between family members and close acquaintances activates socioemotional brain networks (e.g. amygdala and insula) and triggers oxytocin release that, in turn, is suggested to increase social pleasure and have inhibitory effects on sympathetic stress responses (Brown & Brown, 2017). In addition, the “Tend and Befriend” theory postulates that, in case of threat, human beings are prone to respond in a compassionate way (i.e. to affiliate and comfort each other). This compassionate response, in turn, is proposed to associate with an “affiliative neurocircuitry” that triggers oxytocin (i.e. increased caring behavior toward in-group members) and endogenous opioids (i.e. increased responses to social pain and pleasure) (Taylor, 2011).

Regarding previous literature, it appears that experiencing compassion for others may also reduce one’s own stress responses. To date, compassion-based interventions are shown to predict lower psychological stress responses over a 2-week follow-up (Matos et al., 2017), lower self-reported psychological stress responses over a 2-month follow-up (Brito-Pons et al., 2018), and lower self-reported stress reactivity to psychosocial stressors over a 9-month follow-up (Engert et al., 2017). Regarding physiological correlates of stress, compassion-based interventions are found to predict reduced C-reactive protein (CRP) levels over a 6-week follow-up (Pace et al., 2013) and lower inflammatory responses to acute stress after a 6-week follow-up (Pace et al., 2009). Moreover, compassion-based interventions are found to predict other stress-related qualities: for example, reduced worry over a 9-week follow-up (Jazaieri et al., 2014) and decreased negative emotionality after a 1-day-long intervention (Klimecki et al., 2014). Finally, compassion-focused therapy interventions are found to reduce rumination (i.e. to move attentional focus from one’s own worries to the needs of others) (Frostadottir & Dorjee, 2019). Regarding negative affect, however, three intervention studies found that compassion training did not reduce negative affect (Engen & Singer, 2015; Klimecki et al., 2013, 2014).

A major limitation of the compassion-based intervention studies (e.g. loving-kindness meditation), however, is that they provide only partial evidence of compassion’s contribution to dimensions of stress. That is, it remains uncertain whether it is increased compassion or some other element of the intervention that has the alleviating effect on dimensions of stress. A second prominent limitation of the compassion-based intervention studies is that they are not able to elucidate the direction of relationships between compassion and stress in everyday life: that is, whether dimensions of stress could also predict a decrease in compassion. Consequently, it is necessary to investigate the relationship of compassion with dimensions of stress using large population-based datasets with long prospective follow-ups that provide possibilities to control a wider set of covariates and to use such statistical analyses that increase understanding about predictive relationships between compassion and stress.

Many pieces of previous evidence suggest that compassion may predict alterations in stress levels. Specifically, compassionate states and dispositional compassion are related to various biomarkers for stress reactivity (Cosley et al., 2010; Stellar et al., 2015). High compassion may also promote psychological well-being (Saarinen et al., 2019) and protect against the development of hypertension (Saarinen et al., 2020) that is closely related to physiological stress. Furthermore, it is known that compassionate feelings commonly lead to behavioral acts that aim to alleviate others’ suffering such as affiliative behavior (Gilbert, 2017; Gilbert & Mascaro, 2017) that, in turn, can buffer against stress responses (DeVries et al., 2003). Finally, high compassion associates with more favorable health behaviors (Gluschkoff et al., 2018) that, in turn, may enhance stress coping. To the best of our knowledge, no study has investigated the relationship of compassion and vital exhaustion. Regarding negative affect, three studies have found that dispositional compassion for others’ suffering may not associate with negative affect (Arimitsu et al., 2019; López et al., 2018; Medvedev et al., 2020).

Several pathways describing how dimensions of stress might predict lower level of compassion for others have also been proposed. First, some degree of psychological well-being is suggested to be a necessary requirement to be able to feel compassion for others’ suffering. For example, in environments with high levels of stress and life dissatisfaction (e.g. workplace bullying), individuals are more prone to display aggression toward others and to dating violence (e.g. Hauge et al., 2009; Hershcovis et al., 2007; Lewis & Fremouw, 2001). Secondly, stress hormones are found to affect the processing of social cues and to be involved in the initiation of social aggression (Montoya et al., 2012) that, in turn, correlate strongly with a lower level of compassion (García et al., 2012; Lee et al., 2012). Finally, neurobiological studies suggest that stressor exposure may affect the functioning of such brain regions that are responsible for the processing of socioemotional cues and compassionate feelings. For example, exposure to stressors is linked to alterations in the plasticity of amygdala (Roozendaal et al., 2009) and altered opioid release in the nucleus accumbens (Marinelli et al., 2004). Amygdala and nucleus accumbens, in turn, are found to be involved when experiencing compassion for others (Valk et al., 2017; Weng et al., 2013, 2018).

Not only do we lack clarity regarding the direction of the association between compassion and dimensions of stress, we also do not have a good understanding of how stable this association is across different ages. In compassion-based intervention studies, the follow-ups have ranged from a few hours (Abelson et al., 2014) to 9 months (Engert et al., 2017). The non-intervention studies investigating the association between compassion and stress have been cross-sectional (e.g. Cosley et al., 2010; Stellar et al., 2015). Even though cross-sectional studies provide possibilities to investigate the state-level correlations between compassion and stress, prospective follow-ups are vital for investigating the stability of the association between compassion and dimensions of stress across different ages and developmental transitions. There is evidence that the effect of dispositional traits on stress responses may vary over age (Mroczek & Almeida, 2004). To date, it has remained unclear whether the association of compassion with dimensions of stress could change across development or be evident from early adulthood to middle age.

The first aim of the current study was to investigate the predictive relationships of compassion with negative emotionality (i.e. a marker of susceptibility to stress) and vital exhaustion (i.e. a marker of chronic stress responses). That is, whether compassion predicts changes in the levels of negative emotionality and vital exhaustion or vice versa. The second aim was to investigate the effect of compassion on the developmental courses of negative emotionality and vital exhaustion over a long-term prospective follow-up, ranging from early adulthood to middle age. We used population-based data with an over 10-year prospective follow-up and several measurement points. The data also provided possibilities to take into consideration several potential confounders: age, sex, participants’ and their parents socioeconomic factors (level of income and educational level), and agreeableness. On the basis of previous theoretical models (Brown & Brown, 2017; Gilbert, 2019; Kirby & Gilbert, 2017; Taylor, 2011), we had the following research hypotheses: (1) high compassion predicts lower negative emotionality and vital exhaustion over the follow-up, and (2) the predictive pathways are more likely to proceed from high compassion to lower levels of negative emotionality and vital exhaustion than vice versa.

## Methods

### Participants

The participants were members of the prospective longitudinal Young Finns Study. The participants were selected randomly from six birth cohorts (born in 1962, 1965, 1968, 1971, 1974, and 1977) from the population register of the Social Insurance Institution. The original sample included 3596 participants at the baseline measurement in 1980. Since then, eight follow-up measurements have been conducted between 1983 and 2012 (when participants were aged 35‒50 years). The Young Finns Study was carried out in accordance with the Declaration of Helsinki. Additionally, all the Finnish universities with medical schools approved the study design. Before participation, all the participants or their parents (for participants aged below 12 years) provided written informed consent after the nature of the procedures had been fully explained. The design and methods of the Young Finns Study is described more precisely elsewhere (Raitakari et al., 2008).

For the current study, compassion was assessed in 1997, 2001, and 2012; vital exhaustion in 2001, 2007, and 2012; negative emotionality in 1997, 2001, 2007, and 2012; agreeableness in 2007 and 2012; parents’ level of income and educational level in 1980; and participants’ level of income and educational level in 2011. We included in the analyses all the participants who had data available on all study variables in at least one of the measurement times (e.g. data on vital exhaustion in 2001, 2007, or 2012; data on negative emotionality in 1997, 2001, 2007, or 2012; data on compassion in 1997, 2001, or 2012; data on parents’ socioeconomic factors in 1980; data on participants’ socioeconomic factors in 2011). The final data included 1031–1495 participants. The study design is illustrated in Supplementary Table 1.

Attrition was examined by comparing the study variables between the included (*n* = 1573) and excluded (*n* = 2023) participants. Included participants (vs. excluded participants) had slightly lower levels of negative emotionality and vital exhaustion, were slightly older, and had more likely high educational level in adulthood and more likely parents with high educational level. Women were more likely to participate than men. There was no attrition bias in compassion. The results of these attrition analyses are reported with more detail in the Supplementary Table 3.

### Measures

#### Compassion

*Compassion* was evaluated with the Compassion scale of the Temperament and Character Inventory (TCI) (Cloninger et al., 1994). The Compassion scale (Cronbach’s α = 0.85–0.87 in 1997, 2001, and 2012) consists of 10 self-rating items (e.g. ‘It gives me pleasure to see my enemies suffer’ [reverse scored]; “It gives me pleasure to help others, even if they have treated me badly”). The items are responded with a 5-point Likert-scale (1 = completely disagree; 5 = completely agree). The correlations between the single items and the mean score of compassion were significant in all measurement years (r = 0.48–0.81 in 1997; r = 0.47–0.80 in 2001; and r = 0.43–0.76 in 2012). For each measurement time, we calculated the mean score of compassion for all the participants who had responded to at least 50% of the items. There were no statistical differences between complete vs. incomplete responders of compassion in negative emotionality or vital exhaustion in any measurement year. The validity of the Compassion scale has been described with more details elsewhere (see Saarinen et al., 2019).

#### Stress-related dimensions

*Negative emotionality* was measured with the Negative Emotionality scale of the Emotionality, Activity, and Sociability Temperament Survey (EAS) (Buss, 1991). The scale consists of 12 items (e.g. “I’m easily frightened”; “I’m irritated a great deal more than people are aware of”) that are responded with a 5-point scale (1 = totally disagree; 5 = totally agree). In our data, the internal consistency of the scale was good (Cronbach’s α = 0.80–0.82 in 1997, 2001, 2007, and 2012). The correlations between single items and the mean score of negative emotionality were significant in all measurement years (r = 0.37–0.72 in 1997; r = 0.39–0.69 in 2001; r = 0.31–0.71 in 2007; and r = 0.35–0.71 in 2012). We calculated the mean score of the items for all the participants who had responded to at least 50% of the items.

*Vital exhaustion* was measured with The Maastricht Vital Exhaustion Questionnaire (Appels et al., 1987) that includes 21 items (e.g. “Do you ever wake up with a feeling of exhaustion and fatigue?”; “Do little things irritate you more lately then used do?”; “Do you feel you want to give up trying?”) that are responded with a 3-point scale (0 = no; 1 = I’m not sure; 2 = yes). We calculated the mean score of the items for all the participants who had responded to at least 50% of the items. In our data, the internal consistency of the scale was good (Cronbach’s α = 0.88–0.89 in 2001, 2007, and 2012). The correlations between the mean score and single items of vital exhaustion were statistically significant and comparatively strong in all measurement years(r = 0.41–72 in 2001; r = 0.44–0.76 in 2007; and r = 0.44–0.74 in 2012).

Using factor analysis, we examined whether the items of the Maastricht Vital Exhaustion Questionnaire (MVEQ) and the Negative Emotionality scale of the Emotionality, Activity, and Sociability Temperament Survey (EAS) are loaded to different factors. We conducted factor analysis with two factors and Promax rotation. The results of the factor analysis are presented in Supplementary Table 2. The results showed that, except for two cross-loadings (the items 11 and 17 of the MVEQ), the items of the two questionnaires were loaded to separate factors. This indicated that vital exhaustion and negative emotionality have only minor conceptual overlap. In addition, test–retest correlations showed that negative emotionality was comparatively stable over the follow-up [r(1997–2001) = 0.68; r(2001–2007) = 0.68; r(2007–2012) = 0.73; r(1997–2012) = 0.56]. Test–retest correlations of vital exhaustion were somewhat lower for vital exhaustion [r(2001–2007) = 0.55; r(2007–2012) = 0.63; r(2001–2012) = 0.50] than for negative emotionality. This suggested that vital exhaustion may measure a somewhat varying but still fairly long-standing state of chronic stress whereas negative emotionality may refer to a more stable disposition to stress responses.

#### Covariates

Covariates included age, sex, participants’ and their parents’ socioeconomic factors, and agreeableness in adulthood. *Participants’ and their parents’ socioeconomic factors* included education level and level of income. Parents’ and participants’ educational level was classified into 3 categories (1 = comprehensive school, i.e. the first 9 years of school; 2 = high school or occupational school; 3 = academic, i.e. university or college). In case mother’s and father’s educational levels were different, we used the higher educational level. Parents’ level of income was classified into three categories (1 = low; 2 = average; 3 = high). Participants’ level of income was assessed with a 13-point scale (1 = less than 5000€ per year; 13 = more than 60,000€ per year). The socioeconomic factors were included in the analyses as separate variables.

*Agreeableness* was measured with the Finnish version of the Neuroticism, Extraversion, Openness, Five‐Factor Inventory (NEO‐FFI) (see Rantanen et al., 2007). The agreeableness scale included 12 self-rating statements (e.g. “I would rather cooperate than compete with others”) with a 5-point response scale (1 = strongly disagree; 5 = strongly agree). All the items were reversed so that higher values referred to higher agreeableness. The internal consistency of the scale was good (Cronbach’s α = 0.79–0.80 in 2007 and 2012). For each measurement time, we calculated the mean score of the items for all the participants who had responded to at least 50% of the items. Next, we calculated the mean score of year 2007 and 2012 scores of agreeableness for all the participants with data available on 2007 and/or 2012.

### Statistical analyses

We conducted statistical analyses using the version 16.0 of Stata SE. The predictive relationships of compassion with negative emotionality and vital exhaustion were examined using structural equation models with cross-lagged panel design (MLMV estimation). We used the scores of compassion, negative emotionality, and vital exhaustion in 2001 and 2012. We conducted 4 models: (1) a model including only stability coefficients (the variables at each time point were predicted by the same variables at the previous time point) and covariances between compassion, negative emotionality, and vital exhaustion at each time point; (2) a model that included the predictive coefficients from negative emotionality and vital exhaustion at each time point to compassion at the next timepoint; and (3) a model that included predictive coefficients in the opposite direction (i.e. from compassion at each time point to negative emotionality and vital exhaustion at the next time point); (4) a model with the predictive coefficients in both directions. All the SEM models were controlled for age, sex, participants’ and their parents’ socioeconomic factors (level of income, educational level). As additional analysis, we included also agreeableness as covariate.

The statistical fit of the models 1‒4 was evaluated by comparing the values of the χ^2^ test of absolute model fit, Comparative Fit Index (CFI), the Root-Mean-Square Error of Approximation (RMSEA), and the Bayesian Information Criterion (BIC). Adequate values of the CFI range over 0.95, and the value of RMSEA should be below 0.06 (Hu & Bentler, 1999). Moreover, lower scores of the χ^2^ test of absolute model fit and the BIC indicate better model-fit (Schreiber et al., 2006). The selection of best-fitting model was done on the basis of the χ^2^ test of absolute model fit (in case the χ^2^ test value between two models was not statistically significantly different, the “more simple” model was selected).

The longitudinal associations of compassion with negative emotionality and vital exhaustion were investigated with growth curve modeling (MLMV estimation). The growth curve models estimate both fixed effects (regression coefficients) and random effects (individual-level variation in the intercept and residual variance, i.e. within-individual variation over the follow-up). In order to reduce potential multicollinearity in the multilevel models, age was centered to the age of the youngest age cohort in the first measurement year of the outcome variable (i.e. with age of 20 years when predicting negative emotionality in 1997–2012, and with the age of 24 years when predicting vital exhaustion in 2001–2012). As previous studies have found curvilinear trends in the course of compassion over age (Hintsanen et al., 2019), we included age-squared in the growth-curve models.

As additional analysis, we included also agreeableness as covariate (in fixed effects). This analysis was conducted because there is previous evidence that compassion for others correlates strongly with agreeableness (but not with other Big Five traits such as neuroticism) (Di Fabio & Saklofske, 2021; Kim et al., 2020; Pommier et al., 2020). Hence, this analysis was conducted to test whether the effect of compassion on dimensions of stress is confounded by agreeableness.

We estimated separate growth curve trajectories for vital exhaustion (in 2001–2012) and negative emotionality (in 1997–2012). We predicted the curve of vital exhaustion by the compassion score of year 2001; and we predicted the curve of negative emotionality by the compassion score of year 1997 (i.e. the first measurement year of the outcome variable). Intercept, age, age-squared, compassion and its age-interactions, gender, and participants’ and their parents’ level of income and educational level were set as fixed effects.

## Results

The means, standard deviations, and frequencies of the study variables are shown in Table 1. Correlation coefficients between the study variables are presented in Supplementary Table 4.

**Table 1:** The means, standard deviations (*SD*), and frequencies of the study variables

Variable (measurement year)	Mean/frequency (%)	SD	Range
Compassion^a^	3.68	0.63	1–5
Vital exhaustion^a^	0.41	0.36	1–5
Negative emotionality^a^	2.57	0.59	1–5
Gender (Female)	885 (56.26)		
Age in 2001	31.66	5.02	24–49
Parental educational level			
Comprehensive school	503 (32.0)		
High school or occupational school	654 (41.6)		
Academic level (university or college)	416 (26.5)		
Parents’ level of income			
Low	388 (24.7)		
Average	824 (52.4)		
High	361 (23.0)		
Participants’ educational level			
Comprehensive school	39 (2.5)		
High school or occupational school	832 (52.9)		
Academic level (university or college)	702 (44.6)		
Participants’ level of income	7.45	3.07	1–13

^a^In Table 1, we reported the year 2001 values of compassion, vital exhaustion, and negative emotionality

**Fig. 1:**
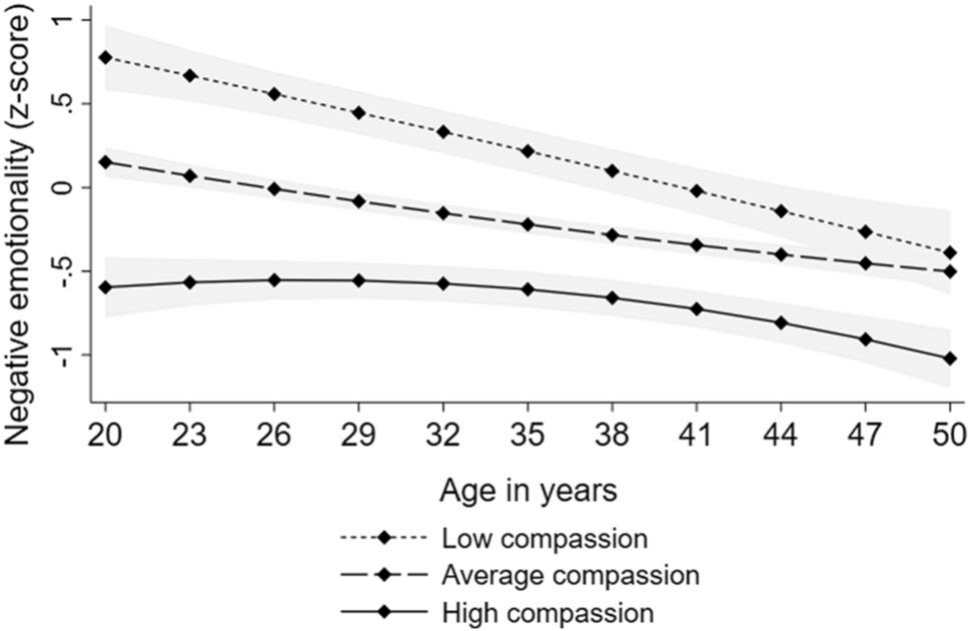
The illustration of Model 3: Standardized predictive pathways from compassion to negative emotionality and vital exhaustion. For clarity, control variables (age, sex and participants’ and their parents’ level of income and educational level) were excluded from this figure. *p < .05, *n* = 1495

The results of the structural equation models are presented in Table 2. The results showed that there was no difference in the statistical fit (*p* = 0.224) between Model 1 (no predictive paths) and Model 2 (predictive paths from negative emotionality and vital exhaustion to compassion). Instead, Model 3 (predictive paths from compassion to negative emotionality and vital exhaustion) had significantly better statistical fit than Model 1 or Model 2 (*p* < 0.00001). There was not significant difference in the statistical fit between Models 3 and 4 (predictive paths in both directions) (*p* = 0.456). Hence, as adding more statistical parameters to the model (i.e. predictive paths in both directions) did not improve statistical fit significantly, Model 3 (with fewer parameters) was selected. Taken together, Model 3 had clearly better statistical fit (RMSEA = 0.043, CFI = 0.979) than Model 1 and 2 and slightly better fit than Model 4. This indicated that the predictive paths from compassion to negative emotionality and vital exhaustion were slightly stronger than in the opposite direction. The predictive relationships (in Model 3) are illustrated in Fig. 1. In Model 3, the predictor variables explained approximately 47% of variance of compassion, 39% of variance of negative emotionality, and 23% of variance of vital exhaustion.

**Table 2:** The goodness-of-fit statistics for the longitudinal models on the predictive relationships of compassion with negative emotionality and vital exhaustion

	χ^2^ value	*df*	*p*	RMSEA	CFI	BIC	Model comparisons		
							χ^2^ difference test	*df*	*p*
Model 1	115.466	24	< .001	0.051	0.969	35,606.369			
Model 2	112.470	22	< .001	0.052	0.969	35,617.994	χ^2^(2 vs. 1) = 2.996	2	0.224
Model 3	82.193	22	< .001	0.043	0.979	35,587.717	χ^2^(3 vs. 1) = 33.273	2	< .00001
Model 4	80.625	20	< .001	0.045	0.979	35,600.768	χ^2^(4 vs. 3) = 1.569	2	0.456

Model 1: No cross-lagged predictive paths

Model 2: Predictive paths from negative emotionality and vital exhaustion to compassion

Model 3: Predictive paths from compassion to negative emotionality and vital exhaustion

Model 4: Predictive paths in both directions

*RMSEA* the root mean square error of approximation, *CFI* the Comparative Fit Index, *BIC* the Bayesian information criterion. *n* = 1495

In the additional analysis, we included agreeableness in adulthood as covariate. The results were largely replicated. That is, Model 3 had higher goodness-of-fit when compared to Model 1 [χ^2^(Model 3 vs. 1) = 33.716, *p* < 0.00001] or Model 2 [χ^2^(Model 3 vs. 1) = 30.036]. Further, adding predictive paths in both directions did not improve the model significantly [χ^2^(Model 3 vs. 1 = 2.116), *p* = 0.347]. Thus, also the additional analysis indicated that there were stronger predictive pathways from high compassion to lower levels of negative emotionality and vital exhaustion than vice versa.

**Fig. 2:**
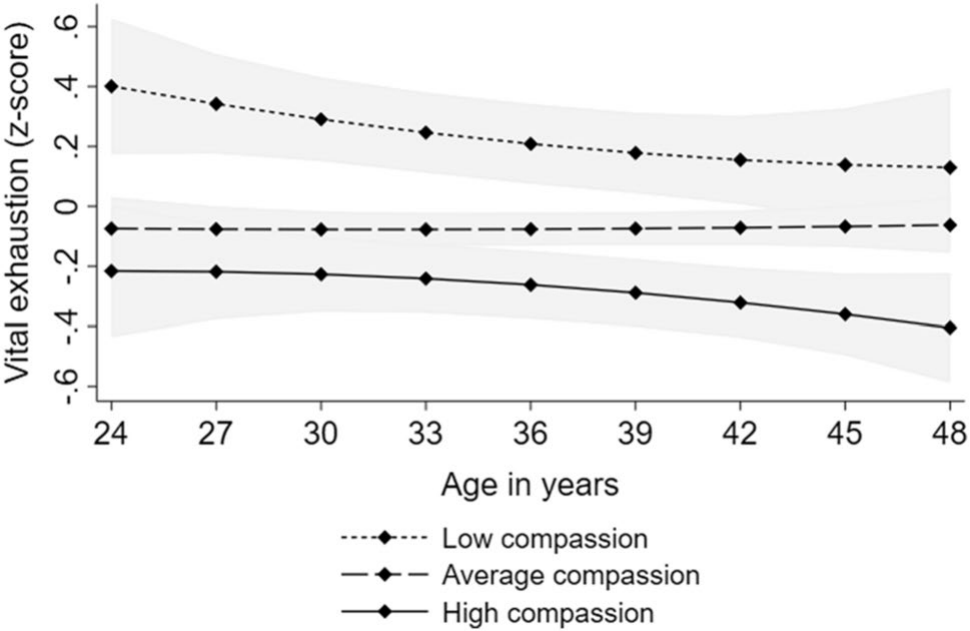
Model-predicted values with 95% confidence intervals (marked with gray color) of (i) vital exhaustion and (ii) negative emotionality over age separately for participants with low (− 1 *SD*), average, and high (+ 1 *SD*) levels of compassion. Adjusted for sex and participants’ and their parents’ level of income and educational level

The results of the growth curve models are presented in Table 3. High compassion predicted lower vital exhaustion. There were no significant interactions between compassion and age or age-squared, indicating that the effect of high compassion on lower vital exhaustion was evident over the whole age range (between the ages of 24–49 years). The results showed that high compassion predicted lower negative emotionality. Further, the interaction between compassion and age was significant, suggesting that the effect of compassion on negative emotionality became weaker over age but was evident from early adulthood to middle age (see Fig. 2). All these findings were adjusted for sex and participants’ and their parents’ level of income and educational level. The findings are illustrated in Fig. 2.

**Table 3:** Results of multilevel models with longitudinal design

	Vital exhaustion (*n* = 1031)		Negative emotionality (*n* = 1212)	
	B	95% CI	B	95% CI
Fixed effects				
Intercept	1.167*	0.672, 1.661	2.675*	2.252, 3.098
Compassion	− 0.273*	− 0.398, − 0.148	− 0.636*	− 0.740, − 0.532
Age	− 0.024	− 0.091, 0.043	− 0.096*	− 0.142, − 0.051
Age squared	0.000	− 0.002, 0.003	0.001	0.000, 0.003
Age*Compassion	0.006	− 0.013, 0.024	0.021*	0.008, 0.033
Age squared*Compassion	0.000	− 0.001, 0.001	0.000	− 0.001, 0.000
Random effects				
Variance of intercept	0.583*	0.549; 0.618	0.649*	0.618; 0.682
Residual variance	0.571*	0.552; 0.589	0.559*	0.545; 0.573

All the models were adjusted for sex and participants’ and their parents’ level of income and educational level

Estimates (B) with 95% confidence intervals (CI) of compassion and age, when predicting standardized scores of negative emotionality and vital exhaustion

*p < .05

In the additional analysis, we included also agreeableness in adulthood as covariate. All the significant associations of compassion with negative emotionality and vital exhaustion remained. The results are shown with more details in Supplementary Table 5.

## Discussion

To the best of our knowledge, this study was the first to investigate the relationship of compassion with stress-related dimensions (i.e. vital exhaustion and negative emotionality) over a long prospective follow-up of over a decade with several measurement points. The results showed that the predictive paths from compassion to negative emotionality and vital exhaustion were significantly stronger than vice versa. This indicates that high compassion is likely to protect against negative emotionality and vital exhaustion, whereas high negative emotionality or high vital exhaustion may not reduce one’s disposition to feel compassion for others over a long-term follow-up. Furthermore, the findings showed that the protective effect of high compassion against negative emotionality and vital exhaustion is evident from early adulthood to middle age.

This study was the first to demonstrate the protective role of compassion against vital exhaustion that is a marker of chronic and repeated stress responses (Keltikangas-Järvinen & Heponiemi, 2004; Koertge et al., 2002; Kudielka et al., 2006). Many theoretical mechanisms have been proposed to explain the pathway from compassionate feelings to lower stress responses. Firstly, by definition, compassion includes an element of non-judgemental attitude toward others’ distress that is postulated to stabilize self-regulation and enhance tolerance also toward one’s own affective states such as distress (Gilbert, 2019; Kirby & Gilbert, 2017). Secondly, it has been emphasized that feelings of social belongingness compose a fundamental evolutionary need for the human being, and lack of compassion may disrupt with maintaining social networks and cause distress via social rejection (Brown & Brown, 2017).

Thirdly, it has been proposed that compassionate inter-dependence between community members (e.g. family members or friendships) activates socioemotional brain networks and triggers oxytocin release that, in turn, is suggested to have inhibitory effects on sympathetic stress responses (Brown & Brown, 2017). This is also in accordance with the “Tend and Befriend” theory (Taylor, 2011). In line with this proposed mechanism, high compassion is related to lower cortisol reactivity, lower heart rate, higher heart rate variability, and a lower risk for hypertension in individuals at familial risk for hypertension (Cosley et al., 2010; Saarinen et al., 2020; Stellar et al., 2015). Hence, compassion may protect against physiological disturbations in the cardiac and neuroendocrinological stress-regulation systems. However, since this study did not include physiological indicators, more research is needed.

A number of previous studies have found that compassion training may not reduce negative affect (Engen & Singer, 2015; Klimecki et al., 2013, 2014) and that dispositional compassion for others’ suffering may not associate with negative affect in questionnaire-based studies (Arimitsu et al., 2019; López et al., 2018; Medvedev et al., 2020). This study, however, demonstrated that high compassion predicts decreased levels of vital exhaustion and negative emotionality over long-term follow-up. The differences between previous findings and our results may be explained by our larger sample size, substantially longer prospective follow-up, and more sophisticated statistical analyses that enabled to differentiate between correlations and predictive pathways between compassion, vital exhaustion, and negative emotionality. Overall, the findings of the current study provide evidence that experiencing compassion for others may not require unstressed psychosocial circumstances but experiencing compassion may reduce stress responses to psychosocial circumstances.

Previously, negative emotionality is found to be a marker of dispositional susceptibility to stress (Strelau & Angleitner, 1991) and to predict higher stress responses at work (Hintsanen et al., 2011) and elevated cortisol levels in response to conflicts (Laurent & Powers, 2007). In this light, the association between high compassion and lower negative emotionality suggests that compassion may predict a higher threshold to experience stress. This may be explained by the findings that compassion is related to better social functioning (Goetz et al., 2010) and may, thus, improve the quality of social relationships. In addition, compassion may protect against stress susceptibility via more adaptive coping strategies. For example, compassion training predicts less frequent use of expressive suppression and higher acceptance when facing psychosocial stressors (Jazaieri et al., 2018). Finally, compassion-focused therapy reduces rumination (e.g. Frostadottir & Dorjee, 2019), indicating that highly compassionate individuals may not be prone to direct their attention to distressing thoughts.

Regarding study limitations, as this was not a genetically informative study, we were not able to study the potential roles of genetic predispositions or gene-environment interactions in driving the association between compassion and stress-related characteristics. It has been found, for example, that oxytocin-receptor genes may affect both empathy and stress reactivity (Rodrigues et al., 2009). Further, the functioning of parental empathy-related brain networks is found to predict offspring’s reactivity to stress (Abraham et al., 2018), implying that there may be heritable aspects in the compassion-stress connection. Longitudinal, multivariate twin data would be able to shed further light on the interaction between compassion and genetic and environmental factors when predicting stress responses.

Secondly, we could not control life events or other environmental factors during the follow-up. On the basis of test–retest correlations, vital exhaustion may change over years to some degree, and it is possible that some favorable life events during the follow-up may alleviate its influence on compassion. Nevertheless, vital exhaustion is commonly regarded as a marker of chronic stress (see e.g. Koertge et al., 2002) and may thus not likely appear or disappear over single exposures to stress. Further, increasing compassion is found to increase also prosocial behavior toward others (Weng et al., 2015) that, in turn, may result in social interactions and situations that are less stressful.

Finally, because this study focused on a developmental period from early adulthood to middle age, it is not able to elucidate how compassion development in early childhood calibrates stress resilience later on. This would be an interesting line of enquiry in the future. To date, there is evidence that children with callous-unemotional traits may exhibit reduced physiological responses to stress (Stadler et al., 2011; von Polier et al., 2013).

This study had also a variety of strengths. Firstly, we had an exceptionally long prospective follow-up: a 15-year follow-up of compassion and negative emotionality and a 10-year follow-up of vital exhaustion. This provided possibilities to investigate the relationship between compassion and stress-related dimensions from early adulthood to middle age. Moreover, we had a fairly large population-based sample. Finally, we used widely used and validated measures of compassion, vital exhaustion, and negative emotionality.

Regarding practical implications, a recent meta-analysis highlighted that interventions for chronic stress (burnout) have focused on the adverse antecedents of stress, whereas positive and preventive factors have remained less investigated (Ahola et al., 2017). Additionally, the meta-analysis pointed out that interventions for chronic stress have most commonly included analyzing one’s mental processes (e.g. cognitive-behavioral therapy, psychodrama, cognitive coping training) and been ineffective for alleviating chronic stress (Ahola et al., 2017). Our findings, in turn, suggest that promoting compassion (i.e. directing attention from the self to others) might protect against vital exhaustion and negative emotionality. Currently, a large proportion of compassion-focused interventions have been provided for individuals in their adolescence or early adulthood (see e.g. Kirby, 2017; Reddy et al., 2013) but our findings provide a promising piece of evidence that increasing compassion training might protect against vital exhaustion also in middle age. There is encouraging evidence that compassion can be cultivated with short-term cost-effective interventions, even within circa 2 months (Jazaieri et al., 2013). Finally, since vital exhaustion is particularly prevalent in individuals with high experienced job demands and low experienced job control (Schuller et al., 2014) and in individuals with work overcommitment (Preckel et al., 2005), there could be also organisation-level interventions enhancing compassion between coworkers in occupational organisations.

## Supplementary Information

Below is the link to the electronic supplementary material.

Supplementary file1 (DOCX 30 kb)
