# Supplementary material for: Compassion protects against vital exhaustion and negative emotionality
Source: Motiv Emot. 2021 Apr 13;45(4):506–17. doi: 10.1007/s11031-021-09878-2 (PMC8550749; doi:10.1007/s11031-021-09878-2)
Supplement: Supplementary file 1 — Supplementary file1 (DOCX 30 kb) [file 11031_2021_9878_MOESM1_ESM.docx]

**Supplementary Table 1.** The study design.

|  | 1980 | 1997 | 2001 | 2007 | 2011/2012 |
| --- | --- | --- | --- | --- | --- |
| Parents’ level of income and educational level | X |  |  |  |  |
| Participants’ level of income and educational level |  |  |  |  | X |
| Compassion |  | X | X |  | X |
| Negative emotionality |  | X | X | X | X |
| Vital exhaustion |  |  | X | X | X |

**Supplementary Table 2.** The results of factor analysis with Promax rotation when including items of Maastrict Vital Exhaustion Questionnaire (MVEQ) and the Negative Emotionality scale of the Emotionality, Activity, and Sociability Temperament Survey (EAS). Note: Factor loadings < 0.3 are not shown for clarity.

|  | Factor 1 | Factor 2 | Uniqueness |
| --- | --- | --- | --- |
| EAS1 |  | 0.439 | 0.806 |
| EAS2 |  | 0.320 | 0.915 |
| EAS3 |  | 0.509 | 0.612 |
| EAS4 |  | 0.399 | 0.806 |
| EAS5 |  | 0.488 | 0.739 |
| EAS6 |  | 0.488 | 0.811 |
| EAS7 |  | 0.361 | 0.807 |
| EAS8 |  | 0.501 | 0.763 |
| EAS9 |  | 0.499 | 0.714 |
| EAS10 |  | 0.664 | 0.552 |
| EAS11 |  | 0.622 | 0.680 |
| EAS12 |  | 0.512 | 0.602 |
| MVEQ1 | 0.355 |  | 0.710 |
| MVEQ2 |  |  | 0.895 |
| MVEQ3 |  |  | 0.870 |
| MVEQ4 | 0.573 |  | 0.582 |
| MVEQ5 | 0.542 |  | 0.647 |
| MVEQ6 | 0.629 |  | 0.572 |
| MVEQ7 | 0.766 |  | 0.486 |
| MVEQ8 | 0.585 |  | 0.577 |
| MVEQ9 | 0.673 |  | 0.522 |
| MVEQ10 | 0.470 |  | 0.769 |
| MVEQ11 |  | 0.347 | 0.739 |
| MVEQ12 | 0.680 |  | 0.601 |
| MVEQ13 | 0.622 |  | 0.612 |
| MVEQ14 | 0.687 |  | 0.497 |
| MVEQ15 | 0.597 |  | 0.716 |
| MVEQ16 | 0.611 |  | 0.642 |
| MVEQ17 |  | 0.350 | 0.810 |
| MVEQ18 | 0.460 |  | 0.802 |
| MVEQ19 |  |  | 0.861 |
| MVEQ20 | 0.528 |  | 0.678 |
| MVEQ21 | 0.324 |  | 0.835 |
| *n* = 1573 | | | |

**Supplementary Table 3.** The results of the attrition analyses between included (*n* = 1573) and excluded participants (*n* = 2023).

|  | Mean / Frequency | |  |
| --- | --- | --- | --- |
| Variable (Measurement year) | Included participants | Excluded participants | *p* value of the difference |
| Compassion (2001) | 3.68 | 3.64 | 0.20 |
|  |  |  |  |
| Vital exhaustion (2001) | 0.41 | 0.46 | 0.009 |
| Negative emotionality (2001) | 2.57 | 2.64 | 0.006 |
| Gender (Female) |  |  | < 0.001 |
| Female | 688 | 1076 |  |
| Male | 885 | 947 |  |
| Age (2001) | 31.66 | 31.27 | 0.021 |
| Parental educational level (1980) |  |  | 0.008 |
| Comprehensive school | 503 | 725 |  |
| High school or occupational school | 654 | 774 |  |
| Academic level (university or college) | 416 | 468 |  |
| Parents’ level of income (1980) |  |  | < 0.001 |
| Low | 388 | 562 |  |
| Average | 824 | 964 |  |
| High | 361 | 354 |  |
| Participants’ educational level (2011) |  |  | < 0.001 |
| Comprehensive school | 39 | 471 |  |
| High school or occupational school | 832 | 529 |  |
| Academic level (university or college) | 702 | 92 |  |
| Participants’ level of income (2011) | 7.45 | 7.03 | 0.020 |

**Supplementary Table 4.** Pairwise correlation coefficients between the study variables.

|  | 1. | 2. | 3. | 4. | 5. | 6. | 7. | 8. | 9. |
| --- | --- | --- | --- | --- | --- | --- | --- | --- | --- |
| 1. Age |  |  |  |  |  |  |  |  |  |
| 2. Sex | 0.012 |  |  |  |  |  |  |  |  |
| 3. Participants' educational level | -0.164* | 0.054* |  |  |  |  |  |  |  |
| 4. Participants' level of income | 0.041 | -0.291* | 0.306* |  |  |  |  |  |  |
| 5. Parents' educational level | -0.270* | -0.044 | 0.284* | 0.143* |  |  |  |  |  |
| 6. Parents' level of income | -0.025 | -0.020 | 0.205* | 0.168* | 0.470* |  |  |  |  |
| 7. Compassion | 0.045 | 0.141* | 0.057* | 0.036 | 0.006 | 0.040 |  |  |  |
| 8. Vital exhaustion | 0.011 | 0.169* | -0.080* | -0.218* | -0.057* | 0.006* | -0.200* |  |  |
| 9. Negative emotionality | -0.033 | 0.247* | -0.095* | -0.239* | -0.053 | -0.057* | -0.320* | 0.561* |  |
| * Statistically significant at significance level of *p* < .05. *n* = 1573 | | | | | | | | | |

**Supplementary Table 5.** Results of multilevel models with longitudinal design. Estimates (B) with 95% confidence intervals (CI) of compassion and age, when predicting standardized scores of negative emotionality and vital exhaustion.

|  | Vital exhaustion (*n* = 993) | |  | Negative emotionality (*n* = 1131) | |
| --- | --- | --- | --- | --- | --- |
|  | B | 95% CI |  | B | 95% CI |
| Fixed effects |  |  |  |  |  |
| Intercept | 2.102* | 1.545, 2.659 |  | 4.064* | 3.580, 4.549 |
| Compassion | -0.163* | -0.291, -0.034 |  | -0.457* | -0565, -0.349 |
| Age | -0.023 | -0.091, 0.045 |  | -0.099* | -0.145, -0.053 |
| Age squared | 0.000 | -0.002, 0.003 |  | 0.001 | 0.000, 0.003 |
| Age*Compassion | 0.006 | -0.013, 0.024 |  | 0.022* | 0.009, 0.034 |
| Age squared*Compassion | 0.000 | -0.001, 0.001 |  | 0.000 | -0.001, 0.000 |
|  |  |  |  |  |  |
| Random effects |  |  |  |  |  |
| Variance of intercept | 0.557* | 0.524, 0.592 |  | 0.603* | 0.572, 0.635 |
| Residual variance | 0.570* | 0.552, 0.589 |  | 0.558* | 0.544, 0.572 |
| * *p* < .05  *Note:* All the models were adjusted for sex, participants’ and their parents’ level of income and educational level, and agreeableness in adulthood. | | | | | |
